# Exact distinction of excitatory and inhibitory neurons in neural networks: a study with GFP-GAD67 neurons optically and electrophysiologically recognized on multielectrode arrays

**DOI:** 10.3389/fncir.2012.00063

**Published:** 2012-09-06

**Authors:** Andrea Becchetti, Francesca Gullo, Giuseppe Bruno, Elena Dossi, Marzia Lecchi, Enzo Wanke

**Affiliations:** Department of Biotechnologies and Biosciences, University of Milano-BicoccaMilan, Italy

**Keywords:** fano factor, firing, mouse, neocortex, multi-site recording, MEA, spikes

## Abstract

Distinguishing excitatory from inhibitory neurons with multielectrode array (MEA) recordings is a serious experimental challenge. The current methods, developed *in vitro*, mostly rely on spike waveform analysis. These however often display poor resolution and may produce errors caused by the variability of spike amplitudes and neuron shapes. Recent recordings in human brain suggest that the spike waveform features correlate with time-domain statistics such as spiking rate, autocorrelation, and coefficient of variation. However, no precise criteria are available to exactly assign identified units to specific neuronal types, either *in vivo* or *in vitro*. To solve this problem, we combined MEA recording with fluorescence imaging of neocortical cultures from mice expressing green fluorescent protein (GFP) in GABAergic cells. In this way, we could sort out “authentic excitatory neurons” (AENs) and “authentic inhibitory neurons” (AINs). We thus characterized 1275 units (from 405 electrodes, *n* = 10 experiments), based on autocorrelation, burst length, spike number (SN), spiking rate, squared coefficient of variation, and Fano factor (FF) (the ratio between spike-count variance and mean). These metrics differed by about one order of magnitude between AINs and AENs. In particular, the FF turned out to provide a firing code which exactly (no overlap) recognizes excitatory and inhibitory units. The difference in FF between all of the identified AEN and AIN groups was highly significant (*p* < 10^−8^, ANOVA *post-hoc* Tukey test). Our results indicate a statistical metric-based approach to distinguish excitatory from inhibitory neurons independently from the spike width.

## Introduction

Information processing in the cerebral cortex depends on complex interaction of many classes of neurons, whose interplay is poorly understood. The recent multisite extracellular recording techniques sample the activity of large neuronal populations. These methods promise to considerably advance our understanding of the basic rules that govern the neocortical circuits. However, several factors blur the identification of specific neuronal types based on the spiking features recorded extracellularly. Even the mere distinction of pyramidal cells from interneurons is all but trivial. For example, during motion discrimination tasks, neurons in the monkey prefrontal cortex are strongly modulated by the behavioral context and the large trial-to-trial spiking variability often masks the correct assignment of spikes to neuronal types (Hussar and Pasternak, 2009, 2010; Churchland et al., 2010; Qi and Constantinidis, 2012).

Several laboratories have sought to distinguish principal neurons based on their generally longer spike duration and faster decay of the autocorrelation function (ACF; Constantinidis and Goldman-Rakic, 2002; Barthó et al., 2004; Mitchell et al., 2007; Le Van Quyen et al., 2010). However, the relationship between spike duration and cell type does not necessarily hold for individual neurons. Short action potential have been observed in pyramidal neurons named chattering cells (Connors and Gutnick, 1990; Gray and McCormick, 1996; Nowak et al., 2003) as well as in principal cells in the pyramidal tract and the ventral premotor macaque cortex (Vigneswaran et al., 2011). In our previous MEA recording studies in long-term cultures from neonatal murine neocortex, we also found frequent inconsistencies between spike duration and autocorrelograms. Therefore, to cluster excitatory and inhibitory cells, we relied on either autocorrelograms (Gullo et al., 2009, 2010a) or the Fano factor (FF), i.e., the variance to mean ratio of the spike count in a defined time window (Fano, 1947; Tolhurst et al., 1983; Baddeley et al., 1997; Gur et al., 1997; Carandini, 2004; Mitchell et al., 2007; Nawrot et al., 2008; Hussar and Pasternak, 2010; Gullo et al., 2012). Use of FF allowed us to effectively classify units in two clusters, whose ratio was consistent with the typical proportion of pyramidal cells and interneurons, as identified with different methods (de Lima et al., 2007, 2009; Sahara et al., 2012). Moreover, *in vivo* MEA experiments in humans indicate that the differences in spike waveform observed in regular spiking (putative pyramidal) and fast spiking (putative inhibitory) cells are accompanied by different ACF, coefficient of variation (CV) and spiking rate (SR; Peyrache et al., 2012).

However, clear-cut statistical parameters which reliably distinguish excitatory from inhibitory neurons in extracellular multi-array recordings are still lacking. In this paper, we correlated the spikes recorded from single-unit electrodes to the neurochemical nature of the corresponding neurons *in vitro*, which is presently impossible to carry out *in vivo*. We used mice expressing the glutamic acid decarboxylase isoform GAD67 fused with GFP (Tamamaki et al., 2003; Sahara et al., 2012). GAD67 is a GABA-synthesizing enzyme usually co-expressed in GABAergic cells with the isoform GAD65, in variable ratios. The GAD67-GFP knock-in mice have been previously characterized by others. The large majority of the GFP^+^ cells express GABAergic markers (Tamamaki et al., 2003; Ono et al., 2005; Suzuki and Bekkers, 2010). Moreover, patch-clamp recording shows that all GFP^+^ neurons are functionally GABAergic (Suzuki and Bekkers, 2010).

To define diagnostic statistics for excitatory and inhibitory neurons (defined as GFP^+^ cells), we collected data from thousands of units. From each, we analyzed continuous recordings lasting several hours and containing thousands of spikes. After standard spike sorting into Mahalanobis-separated units by principal component analysis (PCA), we computed an array of physiologically relevant statistics (Gullo et al., 2009). The cross-correlation dynamics indicated the occurrence of monosynaptic excitatory and inhibitory pathways similar to those observed *in vivo* in rats and humans (Barthó et al., 2004; Peyrache et al., 2012). Altogether, we found that excitatory and inhibitory neurons were best distinguished based on a “count”code identified by the FF (or a “time” code identified by CV^2^; Nawrot et al., 2008), which is also increasingly used to characterize neuronal firing *in vivo* (Churchland et al., 2010; Hussar and Pasternak, 2010; Truccolo et al., 2011).

## Materials and methods

### Ethical statement

Experiments were carried out according to the Principles of Laboratory Animal Care (directive 86/609/EEC), endorsed by the Ethical Committee of the University of Milano-Bicocca. All efforts were made to minimize the number of animals used.

### Cell cultures

Primary cultures of cortical neurons were prepared from GAD67-GFP mice by using standard procedures (Gullo et al., 2009). Mice were kindly provided by Dr. Gerardo Biella (University of Pavia, Italy), with the written permission of Dr. Y. Yanagawa (Gumma University, Japan). Cerebral cortices (except the hippocampus) were removed from decapitated post-natal mice (P1–P3), cut into 1 mm^3^ pieces, and digested by trypsin (0.15%) and DNAase (10 μg/mL), at 37°C for 20 min. Next, cells were mechanically dissociated and plated at densities of 600–900× 10^3^ cells/mL on MEA dishes pre-coated with polyethyleneimine 0.1% (wt/vol) and laminin 20 μ g/mL. MEA dishes had 30 μm diameter ITO electrodes spaced 200 μm apart (Multichannels System, Germany). After incubating for 3 h, the plating medium was replaced by neurobasal medium with B27 (InVitrogen, Italy), glutamine (1 mM) and basic fibroblast growth factor (10 ng/mL). Cultures were maintained at 37°C in 5% CO_2_, with gas-permeable covers (MEA-MEM; Ala Scientific Instruments, Inc., USA). One-half of the medium volume was replaced every 3 days.

### Identification of GFP^+^ cells

**Figure 1:**
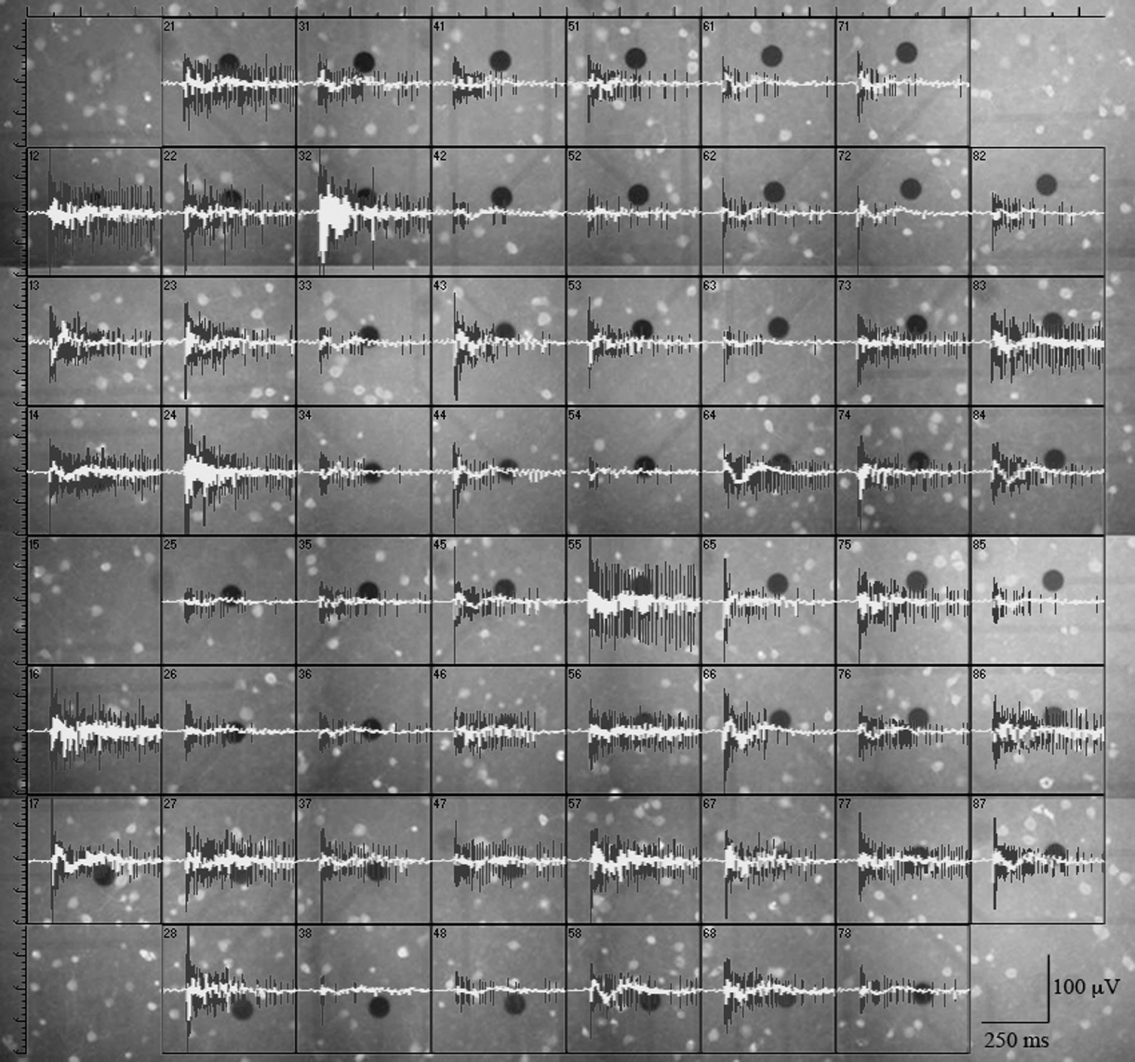
**GFP^+^ cells and burst-related extracellularly recorded traces (spikes and local field potentials) from a representative experiment.** Black circles are metal electrodes (30 μm diameter); fluorescent spots are GFP^+^ neurons; black traces are spike waveforms and white traces are the corresponding local field potentials (see “Materials and Methods”). The distance between adjacent electrodes is 200 μm.

MEA dishes have a recording area of approximately 2 mm^2^, with an average number of neurons (plus glia) in the order of 6000 cells. The average space between cells is therefore relatively wide. Cells were inspected with a Nikon T120 inverted microscope equipped with epifluorescence apparatus and GFP filters (Nital, Italy). The 30 μm diameter circular electrode area appears blind when inspected with inverted microscope (Figure 1), thus masking some of the GFP^+^ cells. However, the 706 μm^2^ electrode area covers less than 10% of the total area sampled by the electrode, whose diameter is about 110 μm. More precisely, fluorescent cells had an average diameter of 9.9 ± 0.08 μm (*n* = 112), in good agreement with the diameter of about 12 μm reported by Ono et al. (2005). Therefore, only cells whose center fell within a 20 μm circle centered inside the 30 μm electrode were totally masked by the metal electrode (assuming an approximately spherical cell shape). Assuming each cell had the same probability to adhere anywhere within the area sampled by the electrode, the probability of a fluorescent cell being fully masked by the electrode was about 3.3%, i.e., the ratio between the 20 μm diameter area and the total sampling area.

### MEA recording; waveform acquisition and sorting

Registrations were carried out at 36°C in in CO_2_-controlled incubators, for no more than 4–5 h per dish, as previously described (Gullo et al., 2009). The entire registration can be thus considered at the steady state (Gullo et al., 2010a). Raw analogue signals sampled at 40 kHz were recorded with MEA-1060BC or 1060INV pre-amplifiers (bandwidth 0.1–8000 Hz; Multichannel Systems), connected to a MEA Workstation (bandwidth 100–8000 Hz; Plexon Inc., USA). To avoid artifacts, the threshold was subsequently readjusted and signals were cleaned of spikes whose inter-spike interval (ISI) was shorter than the pre-fixed 2.5 ms refractory period, by OFFLine Sorter program (Plexon Inc.). Next, during the PCA-based waveform sorting and for multi-unit electrodes, we applied one of the following procedures. (A) Spike removal with a Mahalanobis threshold in the range 1.8–1.4. In this case, we checked that the *p*-value of multivariate ANOVA sorting statistics was <0.01, among the identified units. (B) When the previous procedure led to excessive spike invalidation, we manually removed the spikes invading the adjacent unit ellipsoids. The latter method was very effective in decreasing the *p*-values, even when the number of erased spikes was very low. The units with a spike rate <0.03 Hz, or responding irregularly, or continuously firing during the down-states were discarded. Data shown in Figure 1 were obtained with MC_Rack software (Multichannel Systems), which allows to set the best filtering bandwidth to detect local field potentials (LFP) (5–180 Hz) and spikes (200–5000 Hz; Gullo et al., 2010b). We used 12–15 days-*in vitro* MEA dishes, usually presenting 58–59 active electrodes. The Sorter software acquisition procedure was carried out in a window of 1.2 ms, using a mixed amplitude/duration criterion (Gullo et al., 2009, 2010a).

### Neuronal cluster identification and advanced burst state classification

For each unit, we computed burst duration (BD), spike number (SN), intra-burst spike rate (IBSR), FF (time window of 6 s), ISIs, CV^2^ (computed from ISI histograms), and intra-burst intervals (IBIs). ACF was computed up to 200 ms from the timestamps, with Neuroexplorer. Alternatively (Figure 3), both ACF and the cross-correlation function (CCF) were computed from −50 to 50 ms. Activity bursts were detected and classified as previously reported (Gullo et al., 2009, 2010a, 2012). Briefly, the bursts that presented more than 2 spikes were identified with Neuroexplorer. When two consecutive spikes were observed, we assigned a BD equal to their ISI and a SN of 2. For isolated spikes, we assigned a BD of 3 ms (i.e., larger than the refractory ISI used during acquisition) and a SN of 1. This procedure is based on the following rationale. (1) In all units that sometimes fired a single spike, the large majority of events were bursts containing at least two spikes; consistently, these units always had average SN >2. (2) Pyramidal neurons normally fire few spikes because of feedback and feed-forward inhibitory control (Pouille and Scanziani, 2001). This behavior is typical of CNS neurons and of repeatedly stimulated neurons *in vivo*. Hence, it should also be considered physiological in reverberating networks *in vitro*. Examples of these firing patterns are shown in Figure 4 (upper and middle raster plots). (3) The “classical” burst definition (at least 3 spikes) would have led us to wrongly estimate SN, BD and the burst number. (4) Our networks were silent during the down-states, i.e., the intervals between bursts. We disregarded the units (1–2 in each network) that fired continuously. (5) The effectiveness of these rules was confirmed by the novel type of analysis used in Gullo et al. (2012), in which the concept of “network-burst” was introduced and SN, BD and burst number data well correlated with those used here.

For each neuron, the burst data were averaged over the time segments of interest. Neurons were classified according to an unsupervised learning approach consisting of data reducing PCA, followed by a K-means clustering procedure. Clustering was improved by using an outlier removal procedure that discarded the units whose Mahalanobis distance from the centroid of the cluster was greater than a fixed threshold (we used 1.4). The program generates a series of files associated to the two neuron clusters, giving: (1) the probability density function of finding the 1st, 2nd, 3rd, i-th spikes (firing spike histogram, FSH), which characterizes the neuron firing mode; (2) the FF time-histograms for the time windows of interest. Our software is freely available at: http://boa.unimib.it/handle/10281/25492 (on page bottom, click on right button “apri,” which means “open”). The following file types can be downloaded: source Python code, executable.exe, example ^*^plx files, Origin template files (for graphical purposes that can read ^*^.csv files containing the software output) and explanation text files.

### Statistical data analysis

The data were analyzed and the figures prepared using either OriginPro 7.0 or 8.0 software (OriginLab Co., Northampthon, MA). Data are given as mean values ± S.E.M., with n indicating the number of experiments. Unless otherwise indicated, statistical significance was assessed using a Student's *t*-test at the indicated significance level (*p*).

In Figures 3 and 5, we present a thorough statistical analysis of two experiments in which we recorded, respectively, from AEN (12 units) and AIN (11 units) neurons. These experiments are representative of all our observations.

This is shown in Table 1, in which we present a briefer analysis of the FF properties of the units obtained from 8 experiments that were not illustrated in Figures 3 and 5. In Table 1, the 8 supplementary AEN units are named AEN-sup and the 9 supplementary AIN units are named AIN-sup. Statistical significance between groups was assessed by a one-way ANOVA (*p* < 0.001 with both the *post-hoc* Bonferroni and the Tukey analysis, performed with Origin8Pro). We analyzed FF data from 10 min time segments for each recording. Table 1 indicates that the FF values used in Figures 3 or 5 are not different from those computed from the other experiments (*p* > 0.001). On the contrary, the comparison between the AEN and AIN populations gave a highly significant difference (*p* « 0.001). The Kolmogorov–Smirnov normality test on the data shown in the bottom rows of Table 1 (AEN + AEN-sup and AIN + AIN-sup) showed that the populations were normally distributed (at the 0.05 significance level).

**Table 1:** **Statistical comparison of FF for AEN (Figure 3), AEN-sup, AIN (Figure 5), and AIN-sup**.

	***n***	**mean FF**	**SD**	**SEM**	**Bonferroni**	**Tukey**
AEN (Figure 3)	12	3.17	2.02	0.61	*p* = 0.398	*p* = 0.398
AEN-sup	8	2.54	1.11	0.35		
AIN (Figure 5)	11	27.75	8.45	2.67	*p* = 0.68	*p*= 0.68
AIN-sup	9	29.37	8.81	2.78		
AEN (Figure 3)	12	3.17	2.02	0.61	*p* = 1.4E-8	*p* = 6.3E-8
AIN (Figure 5)	11	27.75	8.45	2.67		
AEN-sup	8	2.54	1.11	0.35	*p* = 1.8E-8	*p* = 1.4E-7
AEN + AEN-sup	20	2.87	1.64	0.36	*p* = 1.8E-16	*p* = 1.9E-8
AIN + AIN-sup	20	28.6	8.6	1.89		

## Results

### Neuronal distribution in the vicinity of MEA electrodes

**Figure 2:**
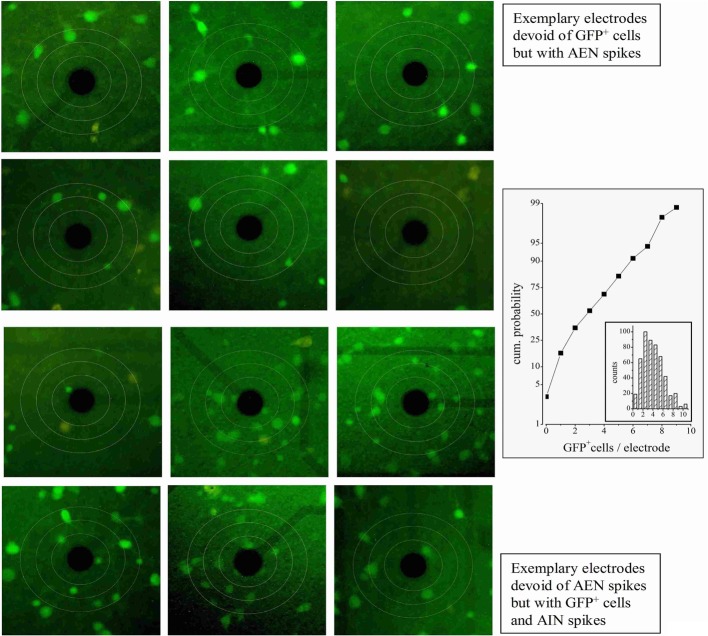
**GFP^+^ neurons near MEA electrodes.** Three circles were drawn centered on each electrode, with diameters of respectively 70, 110, and 150 μm. The upper panels show a series of typical electrodes which showed no nearby GFP^+^ cells within 110 μm. In these cases, all the firing units were found to be of the AEN type. The lower panels show a series of electrodes displaying nearby GFP^+^ cells. In these, all the units were found to be of the AIN type. The plot on the right shows the cumulative probability of finding electrodes with GFP^+^ cells. The inset illustrates the corresponding count histogram obtained from 8 experiments and 1544 cells.

To correlate the spatial distribution of GFP^+^ neurons to the MEA time-series data, we used long-term neocortical networks from neonatal GAD67-GFP mice, with a balanced excitatory-to-inhibitory ratio. We identified 1275 units from 405 electrodes, in 10 independent experiments performed on culture dishes prepared from different mice. Registrations covered about 14 h, at the steady state. A typical experiment is illustrated in Figure 1, in which we associated 60 fluorescent images of different electrode fields with the corresponding spiking trace (black) and LFP (LFP; white). Around each electrode, we outlined concentric circular annuli of 70, 110, and 150 μm diameters. The rare event of having a GFP^+^ cell completely masked by the metal electrode was disregarded (see “Materials and Methods”). Examples of the GFP^+^ cells distribution near the electrodes are shown in Figure 2. For each image, we counted the number of GFP^+^ cells contained within 55 μm from the electrode center. In our experimental conditions, this is the approximate distance beyond which the electrotonic current contribution of each neuron is smaller than the electrode noise (Henze et al., 2000; Pettersen and Einevoll, 2008). A full theoretical analysis is found in Pettersen and Einevoll (2008). Figure 2 shows electrodes surrounded or not by GFP^+^ cells within the sampling area (approximately 9000 μm^2^), as indicated. Overall, 1544 GFP^+^ cells were found within the sampling regions, which correspond to about 2300 μm^2^ per GABAergic neuron, consistent with previous immunocytochemical estimates of around 1000 μm^2^/neuron (Gullo et al., 2010a; recall that the repeated washing procedures of immunocytochemistry cause a 50% cell loss). Such estimate agrees with the results obtained from inferior colliculus slices in central nucleus, dorsal cortex and external cortex, which gave 2800, 6250, and 4150 μm^2^/neuron, respectively (Ono et al., 2005). It is also consistent with the percentages of cortical GABAergic cells observed *in vivo* and *in vitro* (de Lima et al., 2007, 2009).

### Defining the firing properties of AENs

**Figure 3:**
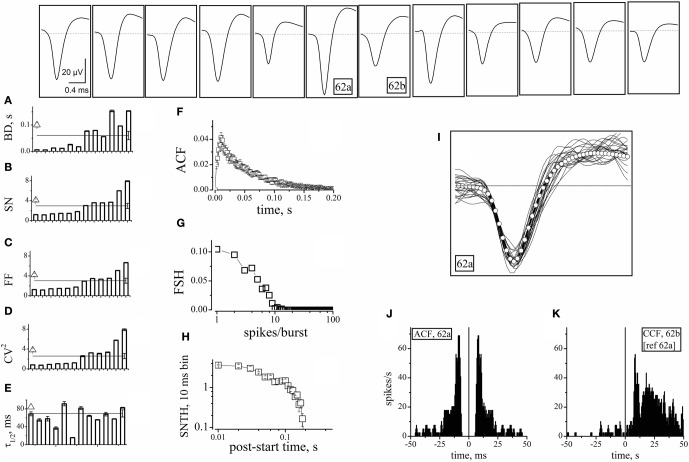
**Properties of units identified from electrodes devoid of GFP^+^ cells.** The upper insets show the averaged (5400 s) waveshapes of spikes assigned to units recorded from electrodes devoid of GFP^+^ cells. **(A–E)** Plots of BD, SN, FF, CV^2^, and τ_1/2_, respectively, computed from data segments of 2 h recordings consisting in ~160 bursts, for each of the 12 units whose spike template is shown in the upward insets. In the same plot, the lines (with error bars at the right end) represent the corresponding mean values. The open triangles shown on the left of panels A–E are average values obtained by using the software mentioned when describing Figure 4. **(F)** The ACF of then 12 units mean. **(G)** The FSH of the 12 units mean. **(H)** The SNTH of the 12 units mean. **(I)** Forty superimposed spikes from unit 62a (lines) and the corresponding mean (open circles) **(J)** ACF of unit 62a. **(K)** CCF of unit 62b, with respect to 62a. ACF and CCF were computed from timestamps. The average SR of units 62a and 62b was 0.033 and 0.062 Hz, respectively. For comparison, SRs of nearby units 64b and 64c (identified as inhibitory cells) were 0.27 and 0.32 Hz, respectively (not shown). The CCF plot shows that, in the 0 to 50 ms region, the conditional probability of observing a spike in 62b given that neuron 62a has fired a spike is ~5 times as high as the probability of observing a spike in the −50 to 0 ms region. For units 62a (in parenthesis unit 62b), during three 1800 s time segments, IBSR was 191, 228, 228 Hz (46, 47, 42 Hz), and FF was 1.82, 1.65, 1.76 (3.4, 3.24, 2.74), respectively.

We first looked for electrodes not sampling GFP^+^ neurons (e.g., top panels of Figure 2). The histogram of the GFP^+^ cell count around our electrodes is shown in the inset to Figure 2 (*n* = 8). Such distribution peaked at 2 and the percentage of electrodes with no GFP^+^ cells was ~3%. In these, the observed spikes had to originate from excitatory neurons. For proper spike assignment to units it is crucial to consider only correct spike waveforms. To this aim, we applied a sorting procedure based first on amplitude (to identify small and large, or close and far away neurons) and then on PCA criteria (see “Materials and Methods”). We thus identified 20 such electrodes (sampling from 20 units) among 405 (a complete ANOVA analysis is given in Table 1). Representative examples are shown in Figure 3, illustrating 12 units characterized in 7 electrodes recording from the same network. Typical waveforms are shown in the upper insets. Recorded traces were analyzed in time segments of ~2 h (Gullo et al., 2009). The bar plots in Figures 3A–E report the indicated firing statistic for each of the units plotted in the upper panels. Although all units arose from excitatory neurons, a large inter-unit variability was observed for BD (CV = 0.94), SN/burst (SN; CV = 0.7) and CV^2^ (CV = 0.85). The data scatter was smaller for the half-time of ACF decay (τ_1/2_; CV = 0.33) and for FF (CV = 0.62).

The SEM values of the intra-unit statistics are given as error bars on each column. These were generally small (and thus sometimes not visible in the plots), because our experimental traces contained, on average, around 160 spontaneous bursts. On the other side, the inter-unit average values for the 12 excitatory neurons were: BD = 0.06 ± 0.016 s, SN = 2.9 ± 0.52, FF = 3 ± 0.5, CV^2^ = 2.6 ± 0.5, and τ_1/2_ = 35 ± 4 ms. These values are reported in Figure 3 as horizontal lines running through panels A–E. The corresponding SEM bars are placed at the right end. They are much larger than the intra-unit ones, because of the relatively small number of averaged units. These results are in overall agreement with literature (Baltz et al., 2010; Gullo et al., 2010a).

It is worth recalling here that cultured neocortical neurons display long silent states (“down-states”) punctuated by briefer “up-states” characterized by frequent firing (Gullo et al., 2010a). The presence of long silent states makes the firing rate an inappropriate measure of the spiking properties of a given neuron. To better characterize the 12 units, we thus also computed the mean ACF (Figure 3F), the frequency of bursts with a given number of spikes (FSH; Figure 3G), and the spike number time histogram, SNTH (Figure 3H; Gullo et al., 2012). The fast ACF decay suggests that these neurons had brief activity during the up-states, and is consistent with the BD and SN values. FSH shows that these neurons were generally unable to produce more than 8–9 spikes per burst. Moreover, SNTH indicates that the number of spikes occurring in 10 ms bins was never higher than 4 and dramatically decreased at the burst end.

To illustrate the spike waveform variability, 40 spikes from the same unit (identified as 62a) and their average (white open circles) were superimposed (Figure 3I). Because the same electrode displayed a second unit (62b), we checked if these cells were connected. We thus computed both the ACF of 62a and the CCF of 62b referred to 62a (Figures 3J–K). The ACF of 62a was very similar to the average data shown in panel F. More interestingly, almost all of the spikes of unit 62b followed within tens of ms those elicited from unit 62a, thus demonstrating an excitatory-to-excitatory connectivity similar to that typically observed in the neocortex.

### Comparing the AEN statistical properties with those defined by a blind software-and FF-dependent clustering

**Figure 4:**
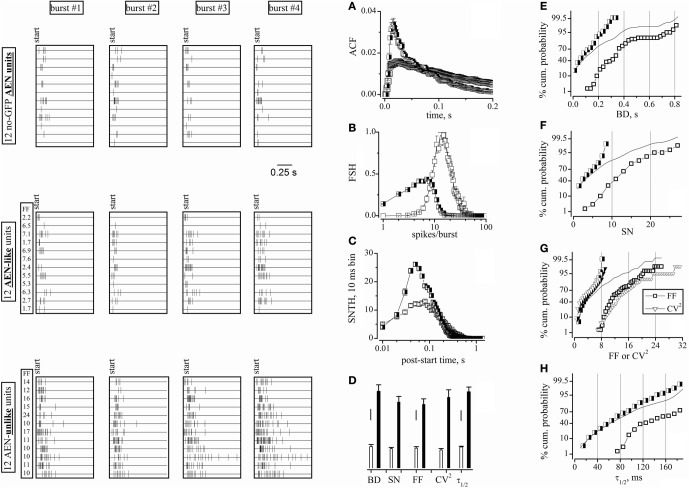
**Raster plots of cell activity and statistical properties of neuron clusters.** Data were obtained from the same experiments and time segments as shown in Figure 3, but using all 98 units. Left panels: four-bursts raster plots for 12 neurons (duration, 1 s). Upper left: spike bursts recorded from electrodes devoid of GFP^+^ cells (the 2nd and 3rd row from top correspond to units 62b and 62a, whose waveforms are shown in Figure 3. Middle-left: spike bursts of the AEN-like type (see the main text). Lower-left: spike bursts of the AEN-unlike type. The left columns labeled FF in the middle and lower panels give the average FF values of each units. **(A–C)** ACF, FSH, and SNTH plots, respectively, obtained by using the software clusterization of the 78 and 20 units classified using FF (time window of 6 s). Half-closed squares: AEN-like units; open squares: AEN-unlike units. **(D)** Bar plots of BD, SN, FF, CV^2^, and τ_1/2_ values computed for the AEN-like (78 units; white bars) and AEN-unlike (20 units; black bars) clusters. Vertical scale bars: BD, 50 ms; SN, FF, and CV^2^, 2; τ_1/2_, 20 ms. **(E–H)** Percentage cumulative histograms of BD, SN, FF-CV^2^, and τ_1/2_, as indicated, for all units (lines), for excitatory (half-closed squares) and inhibitory (open squares) neurons, after FF-based clusterization. The bin width in cumulative histogram were, respectively: 0.025 s, 1, 0.5, 0.5, and 10 ms. For each variable, 3 independent data points were computed every 30 min. Total number of excitatory and inhibitory cells was 234 and 60, respectively.

We next compared the statistics calculated for the above AEN units with those obtained by all the units, regardless of whether they were surrounded by GFP^+^ cells or not. By using the usual FF-based procedure, we obtained two unit clusters that we previously proposed to mainly consist of excitatory and inhibitory neurons (Gullo et al., 2010a). Figure 4 (raster plots) illustrates the spiking pattern of 12 units belonging to the different classes. In particular, the AEN units (top panels) were as described in Figure 3. The AEN-like (middle panels) and AEN-unlike units were randomly extracted from, respectively, the putative excitatory and inhibitory clusters. Each panel shows twelve 1 s-long raster plots of spike timestamps, one for each neuron. Spikes are represented by vertical ticks. Four different bursts are shown for every neuron. Bursts were randomly extracted from a ~1000 s time interval and were aligned for display. The AEN units displayed firing features similar to those observed in the AEN-like units. In fact, the objective software analysis assigned the AEN units to the excitatory cluster, in agreement with the independent observation that the corresponding electrodes were not surrounded by GFP^+^ cells. On the contrary, the AEN-unlike units presented a strikingly different firing pattern, as indicated by the average FF values (given for each cell in the columns labeled “FF”). To further quantify these observations, we plotted the ACF (Figure 4A), the FSH (Figure 4B), and the SNTH (Figure 4C) for AEN-like (half-closed squares) and AEN-unlike (open squares) units. The average values obtained for AEN-like units are given in Figures 3A–E (open triangles on top of the leftmost bars) for immediate comparison with those relative to the AEN units. The statistics extracted from AEN and AEN-like units are very similar. The same applies to the ACF and FSH values (Figures 3F–G).

Figure 4D reports the average values of BD, SN, FF, CV^2^, and τ_1/2_ for the AEN-like (78 units; white bars) and AEN-unlike (20 units; black bars) clusters. Although these values were consistently different between clusters, the intra-cluster heterogeneity might have caused some uncertainty in the FF-based unit assignment. To solve this ambiguity and to clarify the extent of intrinsic data scatter, we computed the complete distribution properties of the above statistics. Figures 4E–H show, for the indicated variables, the percentage cumulative distributions of all units (continuous lines). In no case a clear bimodal distribution was observed. In contrast, the distributions of the FF-based clusters of excitatory (half-closed squares) and inhibitory (open squares) units produced better separated distributions. More specifically, the two distributions significantly overlapped in the case of BD (Figure 4E), SN (Figure 4F) and τ_1/2_ (Figure 4H), but not in the case of FF (and partly CV^2^; Figure 4G). Very similar results were found in all our experiments (not shown), suggesting that an FF-based classification reliably identifies the different clusters. We conclude that the physiologically relevant statistical properties of the units identified in electrodes not sampling from GABAergic neurons are undistinguishable from those presented by the putative excitatory units as defined by the FF-based analysis.

### Defining the firing properties of “authentic” inhibitory neurons (AIN) from AEN-unlike units

**Figure 5:**
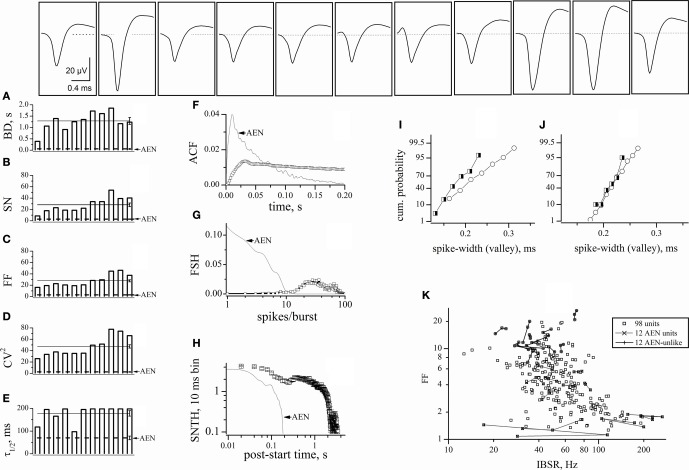
**Characterization of AIN-like units.** Properties of units identified from electrodes sampling from a region containing at least one GFP^+^ cell, but yielding no AEN-like spikes. The upper insets show typical waveforms of the spikes assigned to each unit. **(A–E)** plots of BD, SN, FF, CV^2^, and τ_1/2_, respectively. These were computed from 2 h continuous recordings comprising approximately 160 bursts, for each of the 11 units whose spike templates are shown in the upper insets. In the same plot, the straight continuous lines (with error bars on the right end) represent the corresponding average values for these AINs. For comparison, the dotted lines represent the corresponding values for AEN units, reproduced from Figure 3. **(F)** The ACF of the 11 units mean (open squares). **(G)** The FSH of the 11 units mean (open squares). **(H)** The SNTH of the 11 units mean (open squares; for clarity, only half of the data points are shown). For comparison, dot-lines report the corresponding data calculated from AEN units, reproduced from Figure 3. **(I–J)** Cumulative probability histograms obtained by analyzing the spike-width (at half-maximum amplitude) in the experiments shown in Figures 5 and 3, respectively. Open circles: excitatory neurons; half-filled squares: inhibitory neurons. **(K)** Plot of FF versus IBSR of the 98 units used in the experiment of Figures 3 and 4. For each unit, 3 independent FF (IBSR) data points were computed over consecutive 30 min segments (*n* = 294). To illustrate the data fluctuations during a total 1.5 h recording time, the three data points of each 30-min experiment were connected either by dots- or continuous- lines for, respectively, the 12 AEN and AEN-unlike units (whose timestamps are shown in Figure 4).

After defining the pure AEN properties, we excluded from our analysis all of the traces containing at least one AEN-like unit. We thus also rejected the multi-unit traces containing both AEN-like and AEN-unlike events. Among these electrodes, we selected those displaying the highest number of GFP^+^ cells. We applied this stringent criterion because it has been suggested that the automatic sorting procedure may produce “artifacts” caused by the erroneous sorting of spikes present in the same electrode (Harris et al., 2000). We thus chose one experiment in which we found 29 AEN-unlike units. All of the corresponding electrodes displayed at least 3 GFP^+^ cells. Moreover, 11 units derived from 8 electrodes displaying 5–8 GFP^+^ cells and we found 8 similar units in the other 9 experiments (the ANOVA analysis of all units is given in Table 1). These latter units are analyzed in Figures 5A–E, by using the same statistics as illustrated in Figure 3. Representative waveforms are shown for each unit in the top panels. Overall, the unit variability was relatively low, with CVs in the range 0.2–0.38. On average, these AIN units had BD = 1.29 ± 0.15 s, SN = 28.2 ± 3.2, FF = 27.8 ± 1.8, CV^2 = 47.6 ± 3^ and τ_1/2_ = 89 ± 6 s (continuous lines in panels A–E). For direct comparison, the AEN unit values (dashed lines) were reproduced from Figure 3. The statistics of AEN and AIN units differed by about one order of magnitude.

Once again, for better quantification, we plotted the ACF, FSH, and SNTH graphs (Figures 5F–H). Data from AEN units are given as dotted lines for comparison. The very slow ACF decay suggests that AINs had prolonged activity and is consistent with the BD and SN values. The FSH histogram shows that these cells, differently from AENs, were generally unable to produce fewer than 9–10 spikes/burst. The distribution peaked at approximately 20–30 spikes/burst, consistent with the results shown in Figure 4B. The SNTH plot is a further indication that AINs tended to display prolonged firing, compared to AENs.

### The distribution of the spike-widths was inefficient to sort our units

*In vivo*, the spike-width distribution tends to display a bimodal pattern, with two partially overlapping Gaussian curves attributed to inhibitory (“thin” spikes) and excitatory (“wide” spikes) cells (Constantinidis and Goldman-Rakic, 2002). In agreement with previous observations (Gullo et al., 2009), no such pattern appeared in our data. To deepen the quantitative analysis, we plotted the spike-widths we obtained after software-mediated clustering. Figures 5I and J show the results of analyzing, respectively, the experiments illustrated in Figures 5 and 3. Because the numbers of excitatory (open circles) and inhibitory (half-filled squares) cells grouped by using FF were different, Figure 5 shows the cumulative probabilities instead of the histograms of the counts distributions. Once again, no clear cluster separation is observed. The same analysis performed in other experiments (*n* = 8) gave similar results suggesting that our *in vitro* preparation did not reach, for the spike width, the cell maturation stage observed for the other physiological features we have studied (Gullo et al., 2009, 2010a,b, 2012).

### The FF was unrelated to the intra-burst spike rate

Finally, to investigate if FF was dependent on the typical neuronal firing rate during the up-states, we computed the IBSR for each unit. We studied all the 98 units of the experiment shown in Figure 4, during a period of about 1.5 h, in 30 min time segments (Figure 5K, small open squares). Most of the neurons had a firing frequency between 15 and 200 Hz, which is physiologically reasonable, and no correlation was observed between FF and IBSR. Moreover, for the 12 AEN and AEN-unlike units of Figure 4, the three 30 min data points were connected by a line to allow better appreciation of the typical fluctuations of FF and IBSR values (see legend). These results also show that IBSR is unsuitable for unit clustering.

## Discussion

By using neocortical cultures from GAD67-GFP mice, we localized the GABAergic cells nearby MEA-recording dishes. By collecting data from 405 electrodes, we obtained a sufficient number of electrodes sampling either AENs or GFP^+^ cells and thus distinguished the firing modes of principal cells and interneurons. Although several statistics turned out to be useful to assign neuron types to registered units, the best results were given by FF, which provides a relatively straightforward method to distinguish the two main classes of neurons. Moreover, our work supports the notion that the statistical analysis of spike trains recorded with multi-electrode systems can give reliable information about the neuronal populations. Although combining the optical and electrophysiological analysis *in vivo* is challenging, our results encourage further such studies aimed at dissecting specifically labeled neuronal subtypes.

### Assigning units to neuronal types *In vivo* and *In vitro*

In the hippocampus *in vivo*, Henze et al. (2000) compared the results obtained with extracellular tetrode electrodes and intracellular recording. They found that many intracellular parameters can be inferred by analyzing the extracellular spiking waveforms. In the companion paper, Harris et al. (2000), thoroughly analyzed the possible sources of error in spike clustering for unit recognition. They pointed out the importance of relying on automatic spike-sorting algorithms, instead of human operator choice, for rigorous unit classification. More recently, Barthó et al. (2004) extended multi-unit recording to the somatosensory cortex. They used auto-correlograms to separate units in layer V. Moreover, they showed how to use cross-correlograms to reveal the short-latency synaptic connectivity and thus distinguish pyramidal neurons from interneurons, based on the synaptic effects. In general, however, distinguishing cell type remains difficult, particularly in the neocortex, in which fast spiking does not necessarily identifies interneurons (Connors and Gutnick, 1990; Douglas et al., 1995; Gray and McCormick, 1996; Degenetais et al., 2002; Vigneswaran et al., 2011). Cluster analysis based on physiological and morphological properties leads to indentify several classes of inhibitory cells (Krimer et al., 2005; Zaitsev et al., 2009). Moreover, although working *in vivo* preserves the overall circuitry, it is unclear how deep anesthesia alters neuronal firing and thus unit classification.

A complementary approach is applying MEA recording on long-term neocortical cultures. These allow relatively straightforward identification of specific neuronal types with different labeling methods. Moreover, at the steady state, the balance between neuronal types and the general firing statistics resemble the situation observed *in vivo*. More specifically, the cross-correlogram properties of cultured networks, which indicate the degree of short-latency synaptic connectivity (Gullo et al., 2009), are similar to those observed in sleeping rats (Barthó et al., 2004) and humans (Peyrache et al., 2012). The up-state structure of the reverberating network activity can be dissected by using time-histograms, after unit clustering into putative excitatory and inhibitory neuronal groups (Gullo et al., 2010a). Such clustering fits very well with the data obtained by immunostaining (de Lima et al., 2007, 2009; Gullo et al., 2010a), although it still does not produce a definite classification of units as excitatory or inhibitory. To build on such evidence, we investigated in depth the statistical properties of neuronal types identified by GFP fluorescence. Although we found a relatively wide data scatter in the single unit properties of both AENs and AINs, the corresponding average values of BD, SN, FF, CV^2^, and τ_1/2_ were so different that the probability of erroneous assignments was negligible (*p* < 10^−6^). Such probability was further reduced by outliers' rejection. An example is given in Figure 3C. The FF for the 12 units ranged from 1 to 6, but after the software-based outliers' rejection, the average FF was 3.8 ± 0.3 for the excitatory and 14.9 ± 1.6 for the inhibitory cells. Our analysis program works with user-defined constraints, hence it is always possible to discard units located outside of a proper Mahalanobis distance (Gullo et al., 2009, 2010a). Because spike sorting is normally executed by an electrode-by-electrode analysis (based on PCA or other waveform properties), we would recommend developers of MEA-related software to introduce the possibility of computing the parameters analyzed in the present paper. This would considerably help the users to decide how to assign the units sampled by the same electrode and displaying inadequate PCA separation. These assignments are currently not based on rigorous statistical tools and we suggest that FF is a particularly satisfactory parameter to distinguish excitatory and inhibitory neurons. In agreement with our results, it has been recently observed that, in monkey prefrontal cortex, fast spiking and regular spiking neurons display significantly different FF values (Qi and Constantinidis, 2012).

### Relevance for future studies

As is well known, classifying CNS neurons into broad excitatory and inhibitory populations is a coarse over-simplification. Accordingly, Figures 3 and 5 suggest that, within the main unit clusters, there is ample possibility to define sub-clusters. These further classifications are worth studying in depth, by labeling specific neuronal subtypes. In general, it is unclear whether the unit groups distinguished by firing statistics may be attributed to distinct neuronal types (i.e., characterized by different intrinsic excitability) or simply reflect different synaptic connectivity of otherwise similar neurons. Regardless, our data suggest that spiking heterogeneity is much wider among excitatory neurons. Nonetheless, GABAergic interneurons notoriously comprise many sub-classes defined by gene expression and immunolabeling (Wonders and Anderson, 2006; Suzuki and Bekkers, 2010). Hence, our observations would seem to better fit the idea that the spiking heterogeneity is more related to neuronal connectivity than to intrinsic neuronal variability. A promising approach to further characterize these neuronal populations is studying the correlation between the spikes and the LFP (Gullo et al., 2010b). In the time domain, recent results obtained with 64-electrodes platforms suggest that the temporal heterogeneity can be stable for hours (Gullo et al., 2012). However, the main obstacle to achieve statistical significance when defining unit/neuron subtypes is obtaining a sufficient number of neurons per class in a given experimental record. Only large-scale MEA platforms will permit to gather data from a number of units sufficient to determine the details of such heterogeneity in the space and time domains. Preliminary experiments of ours' with 256-electrodes MEA suggest that the up-states tend not to occur simultaneously between electrodes spaced ~2 mm apart, which agrees with the results obtained *in vivo* in epilepsy studies (Truccolo et al., 2011). Recording with hundreds of electrodes should soon allow to reduce the total number of experiments, when studying the properties of a given CNS region.

Finally, Figures 5I–J indicate that a fraction of inhibitory and excitatory neurons display clearly different spike-widths. Thus the general pattern is similar to the one observed in adult neurons. However, for intermediate spike durations, the separation of neuronal classes in immature networks seems less efficient than it is at later stages. Hence, our results also indicate that the discrimination method we propose may be particularly useful in sorting out principal cells and GABAergic cells before full neocortical maturation. This would greatly facilitate the interpretation of results obtained with multi-unit recording in developing brains. This aspect has potentially important implications for understanding the pathogenesis of the neurologic diseases that present a significant developmental component, such as idiopathic epilepsies (Noebels, 2008).

### Conflict of interest statement

The authors declare that the research was conducted in the absence of any commercial or financial relationships that could be construed as a potential conflict of interest.
